# Supplementary material for: Identification of Fusarium oxysporum f. sp. lactucae Race 1 as the Causal Agent of Lettuce Fusarium Wilt in Greece, Commercial Cultivars’ Susceptibility, and Temporal Expression of Defense-Related Genes
Source: Microorganisms. 2023 Apr 20;11(4):1082. doi: 10.3390/microorganisms11041082 (PMC10142136; doi:10.3390/microorganisms11041082)
Supplement: Supplementary file 1 [file microorganisms-11-01082-s001.zip › microorganisms-2328666-supplementary.pdf]

Table S1. Reaction of lettuce cultivars to isolates associated with the four races of *Fusarium oxysporum* f. sp. *lactucae* and expressed as disease severity index.

Cultivar	Type	Isolate																			
		Race 1				Race 2		Race 3		Race 4				Greek Isolates							
		MAFF 244120 (Japan)		Fus 1.39 (Belgium)		MAFF 244121 (Japan)		MAFF 244122 (Japan)		04750888 (Netherlands)		Fus 1.01 (Belgium)		GTFus1		GTFus2		GTFus3		GTFus4	
Accra	Iceberg	29.6±5.4[a]	PR	32.3±5.7	S	0.0±0.0	R	4.6±2.1	R	11.2±3.3	PR	37.8±6.1	S	52.6±6.5	S	82.1±9.1	HS	62.6±7.9	HS	67.1±8.2	HS
Siberinas	Iceberg	31.2±8.8	S	100.0±0.0	HS	84.7±6.6	HS	5.2±1.1	R	61.3±4.6	HS	29.6±2.1	PR	81.6±4.6	HS	66.8±4.2	HS	58.4±4.4	S	82.6±3.3	HS
Antolina	Butterhead	72.5±3.9	HS	88.8±5.5	HS	53.1±11.2	S	94.2±1.8	HS	78.6±9.1	HS	93.2±0.7	HS	37.2±2.2	S	26.6±5.2	S	19.8±2.6	S	11.6±5.6	S
Sandalina	Butterhead	22.7±4.8	PR	9.8±3.1	R	15.1±3.9	PR	4.9±2.2	R	12.4±3.5	PR	0.0±0.0	R	0.0±0.0	R	0.0±0.0	R	0.0±0.0	R	7.6±1.1	R
Cencibel	Lollo rossa	0.0±0.0	R	40.6±9.8	S	47.2±6.9	S	4.4±0.7	R	9.8±2.1	R	26.3±2.2	PR	91.1±11.2	HS	91.2±9.6	HS	100.0±0.0	HS	84.9±12.1	HS
Lugano	Lollo rossa	11.2±4.3	PR	82.8±11.1	HS	52.3±8.8	S	65.8±4.7	S	91.1±5.4	HS	49.7±9.2	S	94.6±2.3	HS	82.2±8.8	HS	68.6±4.6	HS	87.6±6.1	HS
Grazion	Batavia	6.4±2.5	R	65.6±8.6	HS	0.0±0.0	R	0.0±0.0	R	73.9±11.2	HS	75.2±8.6	HS	44.6±2.3	S	41.9±6.5	S	74.6±2.3	HS	64.9±8.1	HS
Starfighter	Batavia	11.1±5.5	PR	82.4±12.8	HS	0.0±0.0	R	54.2±4.6	S	81.3±9.6	HS	29.6±5.4	PR	6.2±2.1	R	9.2±3.1	R	8.8±2.1	R	7.1±8.4	R
Kireve	Oakleaf	74.6±8.6	S	71.9±8.7	HS	4.6±2.2	R	9.7±3.1	R	6.1±2.5	R	53.8±7.3	S	6.3±2.5	R	16.3±4.0	PR	19.4±4.4	PR	14.9±3.9	PR
Prunai	Oakleaf	48.8±9.1	S	100.0±0.0	HS	35.6±4.2	S	71.1±4.4	HS	96.8±0.6	HS	81.2±4.4	HS	41.2±5.6	S	58.8±4.4	S	48.1±3.3	S	83.6±7.4	HS
Tacitus	Romaine	3.2±0.4	R	9.8±1.8	R	9.8±3.1	R	0.0±0.0	R	21.1±4.6	PR	23.8±8.3	PR	26.4±4.4	PR	25.5±8.7	PR	7.6±1.2	R	13.8±5.5	PR
Tanius	Romaine	11.2±3.3	PR	18.8±4.3	PR	54.4±7.4	S	0.0±0.0	R	17.2±4.1	PR	12.1±3.5	PR	33.6±1.9	S	50.6±7.1	S	62.3±7.9	HS	49.3±4.4	S
Cavolo di Napoli	Loose-leaf	58.7±8.3	S	71.6±5.1	HS	52.6±3.4	S	72.9±3.1	HS	61.4±4.4	HS	42.1±4.4	S	55.6±3.6	S	51.9±3.1	S	54.7±2.3	S	66.8±8.2	HS
Patriot	Iceberg	41.6±5.4	S	78.8±2.8	HS	66.7±5.3	HS	76.4±3.9	HS	52.5±5.5	S	41.8±5.7	S	58.7±5.9	S	53.7±5.3	S	50.0±2.0	S	62.0±3.2	HS
Costa Rica No. 4	Romaine	18.9±5.6	PR	9.3±3.1	R	73.2±3.1	HS	68.9±5.4	HS	88.7±2.2	HS	92.4±0.7	HS	5.6±4.4	R	0.0±0.0	R	0.0±0.0	R	0.0±0.0	R
Banchu Red Fire	Butterhead	49.8±2.9	S	31.6±4.8	S	8.7±4.4	R	38.9±1.9	S	24.7±3.1	PR	25.1±5.4	PR	41.6±4.6	S	53.8±5.4	S	38.8±4.4	S	32.8±3.1	S
Romana Romabella 30 CN	Romaine	8.8±1.1	R	5.4±2.4	R	0.0±0.0	R	78.8±3.1	HS	54.4±4.6	S	56.6±6.6	S	12.9±1.1	PR	11.4±4.5	PR	6.6±0.9	R	0.0±0.0	R

[a] Values expressed as mean disease index scores ± standard deviation. DI scale 0–100: R, resistant=0–10; PR, partially resistant=11–30; S, susceptible=31–60; HS, highly susceptible=61–100.

Table S2. List of primer sequences used for gene expression analysis in real-time quantitative polymerase chain reaction (RT-qPCR) assays.

Primer name	Primer Sequence (5'→3')	Gene	Reference	NCBI ID/ Lettuce ID (Lsa)*
**HPL1.For**<br>**HPL1.Rev**	CGTTAGGATCCGCCGACCGC<br>TCCTTCCTTGCCCGCCCGTA	Fatty acid hydroperoxide lyase (*HPL1*)	[36]	XM_023907774.1
**LTC1.For**<br>**LTC1.Rev**	AACGAGGGATGCCTTAAGCC<br>CCCGGAAAAGTAAACCCATCG	Sesquiterpene synthase (*LTC1*)		AF489964
**SOD.For**<br>**SOD.Rev**	CTTCCAGCCTTCAACAACGC<br>ATTAGGCCTCCAAACGAGCC	Superoxide dismutase (*SOD*)		AJ310450.1
**ERF1.For**<br>**ERF1.Rev**	TCGCCGGTGATGTCCAGTTATCAA<br>TGTTTCCCTCTCTGCTGGTTCACA	Ethylene response factor 1 (*ERF1*)	[35]	Lsa016859.1
**PRB1.For**<br>**PRB1.Rev**	ATGGGACAGTCGTGTGGCTAGTTT<br>TGTTCACAGCATCTACACCGGTCA	Basic pathogenesis-related 1 (*PRB1*)		Lsa018589.1
**PAL1.For**<br>**PAL1.Rev**	TGGCCCCACCGGAGAAGTCC<br>GGAAGCCATCCCGGACCCCA	Phenylalanine ammonia lyase 1 (*PAL1*)		Lsa044239.1
**LOX.For**<br>**LOX.Rev**	GCAACTAAGCGTGCTTCACCCAAT<br>TGCCTCAAGAAGACCTCCACCATT	Lipoxygenase *(LOX*)		Lsa036946.1
**MPK.For**<br>**MPK.Rev**	GGCGGACTTGAACGGAGGAGC<br>AGCGAACGAACTGGCCTCCA	Mitogen-activated protein kinase (*MPK*)		Lsa036022.1
**BG.For**<br>**BG.Rev**	TCAGCCGGTGCATTTGGAGCA<br>ACCCTGTGACGCGTGTGCAA	*β*-glucanase (*BG*)		Lsa037249.1
**GST.For**<br>**GST.Rev**	CCCGGCGGTTGAAGACGGTG<br>ACACTGATTGCTCCGCCGCC	Glutathione S-transferase (*GST*)		Lsa035144.1
**Actin.For**<br>**Actin.Rev**	AACTGGAATGGTGAAGGCTGG<br>TTGTAGAAAGTGTGATGCCA	*Lactuca sativa* putative actin 7 (*Actin*, Reference gene)	[36]	XM_023905463

*Accession numbers of sequences submitted to the National Center for Biotechnology Information (NCBI) and lettuce ID (Lsa).
